# Genomic Prediction of Milk Fat Percentage Among Crossbred Cattle in the Indian Subcontinent

**DOI:** 10.3390/ani15071004

**Published:** 2025-03-31

**Authors:** Raghavendran Vadivel Balasubramanian, Murali Nagarajan, Marimuthu Swaminathan, Raja Angamuthu, Muralidharan Jaganadhan, Saravanan Ramasamy, Malarmathi Muthusamy, Thiruvenkadan Aranganoor Kannan, Sunday Olusola Peters

**Affiliations:** 1 Department of Animal Genetics and Breeding, Veterinary College and Research Institute, Namakkal 637002, Tamil Nadu, India; raghavendran@tnau.ac.in (R.V.B.); saravanan.r@tanuvas.ac.in (S.R.); malarmathi.m@tanuvas.ac.in (M.M.); 2 Alambadi Cattle Breed Research Centre, Tamil Nadu Veterinary and Animal Sciences University, Dharmapuri 635111, Tamil Nadu, India; 3 Central Research Station, BAIF Development Research Foundation, Pune 411058, Maharashtra, India; mswami@baif.org.in; 4 Department of Animal Biotechnology, Madras Veterinary College, Chennai 600007, Tamil Nadu, India; raja.a@tanuvas.ac.in; 5 Mecheri Sheep Research Station, Pottaneri, Salem 636453, Tamil Nadu, India; muralidharan.j@tanuvas.ac.in; 6 College of Poultry Production and Management, Hosur, Mathigiri 635110, Tamil Nadu, India; thiruvenkadan.a.k@tanuvas.ac.in; 7 Department of Animal Science, Berry College, Mount Berry, GA 30149, USA

**Keywords:** GEBV, milk fat percentage, crossbred cattle, smallholder dairy system, genetic improvement

## Abstract

This study focused on improving the milk fat percentage for crossbred dairy cows in smallholder systems, using GEBV. The data were collected from 2507 animals between 2016 and 2023 under BAIF’s Enhanced Genetic Gains program in Pune, India. After refining the dataset, 33,845 records from 1896 animals were analyzed. The result showed that 75.54% of farms had either one or two animals. Prior to quality control, the mean milk fat percentage was 3.94%, but it decreased to 3.83% after data pruning, which necessitated removing the outliers. Genetic analysis involved 1478 animals genotyped for 49,911 SNPs after applying a rigorous quality control process, and imputation improved the accuracy of genomic data, boosting allele frequency correlation from 0.594 to 0.882. The study revealed that the additive genetic variance, phenotypic variance, and error variance were calculated as 0.012, 0.118, and 0.106, respectively. The heritability was estimated at 0.10, suggesting cautious use for breeding improvements. The GEBV ranged from 0.096 to 3.10%, which offers breeders a practical tool for selecting high-fat-producing cows. This research provides valuable insights into optimizing milk quality and advancing genetic improvement strategies in smallholder dairy systems.

## Simple Summary

This study was conducted to improve the milk fat percentage for crossbred dairy cows in smallholder systems, using genomic-estimated breeding value (GEBV). The phenotypic data were collected across six states in India from the crossbred cows with varied level of exotic inheritance. The genetic analysis was carried out using 50k SNP BeadChip, and imputation improved the accuracy of genomic data, boosting allele frequency correlation. The analysis was conducted using FImpute software version 2.2. Heritability was estimated using Bayes R, suggesting cautious use for breeding improvements. The GEBV offers breeders a practical tool for selecting high-fat-yielding cows. This research provides insights into enhancing milk fat percentage and genetic improvement strategies in smallholder dairy systems.

## 1. Introduction

The dairy sector assumes a great deal of significance in India, and more than 80 million people are employed in this industry, the bulk of whom are landless peasants and small-scale and marginal farmers. This industry is a major source of employment, particularly for women, and is at the forefront of women’s empowerment. Millions of people, mostly women, are employed directly or indirectly in the dairy industry. About 70 percent of dairy farmers in India are thought to be smallholders, and they frequently make dairy farming their main source of income. The industry also sustains millions of workers in the transportation, retail, and milk-processing sectors, which has a ripple impact on employment in both urban and rural areas. India’s extensive dairy farming industry has contributed to the country’s notable increase in milk and dairy product production and consumption during the past few decades. Milk production in India is an important component of the agricultural economy, contributing significantly to the rural economy and providing a major source of income, particularly for rural households; it also plays an important role in reducing poverty and improving livelihoods [1,2,3].

India is the world’s largest producer of milk, accounting for 24 percent of global production, and the milk production has increased by more than 61 percent, from 137.7 million tonnes in 2013–2014 to 221.1 million tonnes in 2021–2022. Furthermore, per capita milk availability has increased from 303 g/day in 2013–2014 to 444 g/day in 2021–2022, an almost 1.5-fold increase [4]. Rich in proteins, vitamins, and minerals, milk is an essential component of the Indian diet, and millions of people, especially children, depend on it for vital nutrition; additionally, it is a crucial element of India’s traditional eating customs. The expanding middle class and urbanization are driving up demand for milk and dairy products. In general, milk production in India is not just an economic powerhouse, but also an integral part of the country’s nutritional, cultural, and social fabric [5].

India’s leading milk production owes to the strategic use of crossbred animals and the invaluable contributions of the smallholder dairy system. In the realm of dairy farming, one pivotal factor that directly influences the composition and overall quality of milk is its fat percentage. Milk fat percentage is an important factor in dairy production since it affects the economic value, processing quality, and nutritional properties of milk. The fat content of milk is critical in evaluating its viability for dairy product processing, especially high-value goods such as butter, cheese, and cream. For example, cheese yield is linked to milk fat and protein levels; hence, dairy processors prefer higher-fat milk. Furthermore, in many milk payment systems across the world, as well as in India, farmers receive larger premiums for milk with higher fat content, which increases the profitability of dairy operations. Therefore, both large-scale dairy enterprises and smallholder farmers looking to increase their profits must optimize the milk fat content. Since smallholder dairy farmers frequently sell their milk directly to customers or through unofficial marketplaces where consumers favor richer, creamier milk, the amount of milk fat is especially important to them. Although they produce less milk overall, breeds like Jersey and native cattle are favored over Holsteins in many developing nations, including India, due to their higher milk fat content [1,2,6].

The feeding practices also play a critical role in maintaining or boosting milk fat levels. Providing high-quality forage, boosting fiber intake, and adding energy-rich feeds like oilseeds might help smallholder farmers to raise milk fat percentage, hence enhancing market value and consumer demand for their milk. Milk fat is important for nutrition in addition to its economic benefits. It is an important source of vital fatty acids, fat-soluble vitamins (A, D, E, and K), and energy, especially for communities who rely largely on dairy products for nutrition [7]. Optimizing milk fat content helps to improve food security and dietary quality in smallholder systems, where dairy is both a source of income and household nutrition. As a result, increasing milk fat content through improved genetics, feeding, and management approaches can benefit both farmers and customers in a variety of dairy systems [8].

Previous research on milk fat percentage has mostly focused on phenotypic and environmental factors, with little emphasis on genomic selection in Indian crossbreeds. Although genomic prediction methods have proved successful in purebred dairy populations in industrialized nations [9,10], their relevance to Indian crossbreeds is unknown due to genetic variations and environmental variables. Studies on indigenous and crossbred cattle in India have revealed significant genetic diversity in milk composition features [11,12]; however, the use of genomic methods to improve milk fat percentage has not been thoroughly investigated. Furthermore, most genomic studies in India have used relatively limited datasets and traditional statistical methodologies, limiting their predictive accuracy [13,14].

Crossbred dairy cattle, which are largely a combination of indigenous breeds and high-yielding exotic breeds such as Holstein Friesian and Jersey, play an important role in milk production in India. However, genetic improvement for milk fat percentage in these crossbreds has primarily relied on traditional selection methods rather than genomic predictions. Given India’s expanding demand for high-fat milk products, it is critical to develop reliable genomic selection models that are tailored to the particular genetic makeup of Indian crossbreds [15]. Implementing crossbreeding programs continuously since the late nineteenth century has created difficulties in selecting replacement heifers with varying levels of exotic inheritance for genetic improvement. Animal breeding is typically subject to human selection based on how well the animal performs across a wide range of qualities. However, conventional selection methods do not hold well for selecting the replacement heifer because of the small herd size, longer generation interval, and uncertainty in phenotypic records. Any genetic improvement program implementation in a crossbred population has the primary hurdle of a small livestock population being reared by small, marginal farmers and landless laborers. Secondary hindrance accounts for varied production environments that will hamper the accuracy of the breeding value of the animals. The third major constraint is the funding for recording the phenotypic data in a wider environment and the enormous time consumption to estimate the breeding value using the conventional accumulated pedigreed performance data [16].

Genomic prediction has emerged as an effective method in animal breeding, allowing for the more precise and efficient selection of desirable features. In dairy cattle, milk fat content is an important economic feature that influences both milk quality and market value. While substantial progress has been achieved in the genetic prediction of milk fat content in purebred dairy cattle, notably in breeds such as Holstein and Jersey, there is still a significant gap in research that focuses on crossbred cattle, particularly in the Indian subcontinent [10,15]. Unlike conventional selection, genomic selection takes advantage of both the phenotypic and genotypic data of the targeted population in predicting the breeding value. By utilizing the genomic information, the breeding value of the animal can be estimated with more accuracy than before in a shorter period. This will help to produce faster genetic gain as the generation interval greatly reduces. For traits with low heritability, sex-limited traits, and traits related to fertility, the efficiency of genomic selection is high compared to traditional methods. This not only simplifies the selection process but also limits the inbreeding trends. Therefore, whole-genomic selection is the effective modern breeding method for the production and selection of superior animals [17,18,19].

The identification of single-nucleotide polymorphism (SNP) markers has opened the way for studying the quantitative trait loci (QTL) linked with specific phenotypes and utilizing them as a tool for selection. The SNPs as markers enable all QTLs in the genome to be identified indirectly from the mapping of chromosome segments defined by their adjacent marker [20]. In this context, the crossbred animals will be genotyped for SNP markers in the genome using either 50K medium-sized SNP chips or highly dense 770K SNP chips. The next step is estimating all SNP effects in a set of reference populations that consists of individuals with phenotypic records and genotypes. An increase in the number of animals in the reference population will in turn increase the accuracy of the prediction equation. Finally, the genomic selection ends with the prediction of genomic breeding value for selection candidates with no phenotypes recorded of their own [21]. The genomic selection process has been significantly improved by the availability of high-density single-nucleotide polymorphism (SNP) markers. Genome-wide association studies (GWASs) have made it feasible to pinpoint genomic areas linked to desirable qualities for subsequent selection. Linkage disequilibrium (LD) between SNPs and the causative variations is necessary for this research, which implies that both genotype and phenotypic data must be available. An admixed population’s LD between SNP markers and causal variants may result from LD inherited from the parent population, which is also created during population crossing. Additionally, GWASs aid in the discovery of putative candidate genes linked to resilience and economic characteristics. Such approaches were used by a number of researchers to find candidate genes and selection signatures [22,23,24].

The phenotypic quality control is a crucial precursor to genomic analysis, as it paves the way for a more comprehensive understanding of the genetic factors governing variations in milk fat percentage. This, in turn, aids in the enhancement of breeding programs by promoting desirable traits [25]. This preliminary analysis is instrumental in the identification of outliers—data points that significantly deviate from the overall distribution [26]. Moreover, the elimination of outliers contributes to the refinement of statistical models used for genomic analysis, yielding predictions of breeding values that are more robust and dependable [27]. Genotypic quality control also plays a pivotal role in modern genetic research and clinical applications, ensuring the reliability and accuracy of genetic data [28]. In the era of genomics, where vast amounts of genomic information are generated, it is essential to maintain the highest standards of data quality to draw meaningful conclusions and make informed decisions in revolutionizing the breeding program [29,30].

The livestock production system in developing countries mostly depends on smallholders, which necessitates the implementation of newer technologies for fetching faster genetic improvement. In India, Bharathi Agro-Industrial Foundation (BAIF) Development Research is a significant non-governmental organization that provides livestock development services to over 2.94 million farm households. The crossbred cows, as well as crossbred and purebred exotic bulls in the artificial insemination stud, are genotyped as part of BAIF’s extensive smallholder dairy recording program, spanning six states in India, to produce estimates of breed composition and genomic-estimated breeding value (GEBV) [30,31].

The existing genomic prediction models often fail to account for the unique genetic diversity and population structure of crossbred cattle, leading to suboptimal prediction accuracy. This gap is particularly significant in the Indian context, where the genetic heterogeneity of crossbred populations is influenced by the admixture of multiple breeds, each contributing distinct genetic variants. Additionally, the lack of large-scale genomic datasets specific to Indian crossbred cattle further complicates the development of robust prediction models. This study aims to address these knowledge gaps by developing a genomic prediction model specifically tailored for milk fat percentage in crossbred cattle in the Indian subcontinent. This study aims to provide insights into the genetic basis of milk fat percentage in crossbred populations and develop tailored prediction models that can inform breeding decisions, thereby enhancing the productivity and sustainability of the Indian dairy sector.

## 2. Materials and Methods

The milk fat percentage data used in this study were obtained from the Enhanced Genetic Gains program of BAIF, Pune. Phenotypic data were collected from smallholder dairy systems with Holstein Friesian or Jersey Crossbreds cows in six states between May 2016 and March 2023. The dataset encompassed 2507 animals from 1659 farms under 75 Community development centers (CDC), resulting in a total of 45,978 records of fat percentage measurements, which were analyzed using an electronic milk tester. All the animal records were given auto-generated unique identification numbers in an admixture of alphanumeric characters auto-generated by the server. The genotypic data were collected from the server in batches that were genotyped with the GeneSeek Genomic Profiler (GGP) Bovine 50K BeadChip (Neogen GeneSeek Operations, Lincoln, NE, USA). The collected data were subjected to various phenotypic and genotypic quality control processes before attempting the analysis.

**Figure 1:**
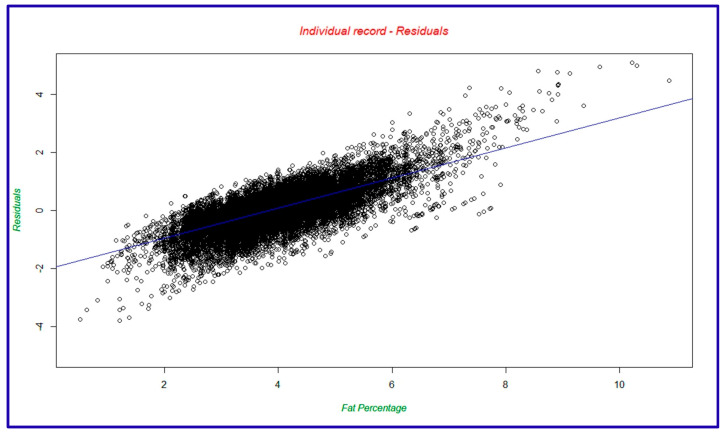
Residual plot for individual animal’s milk fat percentage.

Data were obtained from a total of 2507 crossbred animals. The removal of outliers in the data played a vital role in arriving at a data structure more suitable for analysis. The sequential steps were modified from the steps detailed by Al Kalaldeh et al. [31] for milk yield data:

1. Retention of cows with four or more monthly records taken between 8 and 340 days after calving on milk fat percentages for each lactation;
2. Removal of cows with an average milk fat percentage greater than 3 standard deviations above the population average;
3. Based on an initial statistical analysis using a linear regression model by R script, the removal of individual records for which the standardized residual was outside the −2 to +2 range (Figure 1).

The genotyping of the animals for all the 50K single nucleotide polymorphisms (SNPs) was performed using Genome Studio 2.0 software, based on the raw scan data [32]. The genotype data of 1477 crossbred animals were studied. The autosomal SNPs with a Gen Call (GC) score higher than 0.15 with a call rate higher than 0.9 for each sample ID, higher than 0.95 for each SNPs, and a Minor Allele Frequency (MAF) higher than 0.01 were retained at the time of genotypic quality control [31]. Duplicate samples were identified based on a correlation between genotypes higher than 0.98. A call rate of 0.9 is important to help to minimize the impact of missing data on the analysis and to ensure accurate prediction in genomic selection. Lower call rates can introduce noise and reduce the statistical power of the models used. Low call rates can also lead to biased estimates of genetic effects. All the steps were performed only over the Mobaxtreme application connected with the BAIF server, as this involves enormous memory usage for complex genotype data. Imputation to 770K HD genotype was performed using FImpute software version 2.2 [33].

The phenotypic and genotypic analysis was performed using an animal repeatability model, considering both the random effect of animals and herds, to generate the adjusted test-day milk fat percentage records, assuming that all the animals were not related to each other.

***y*** = ***Xb*** + ***Z***_1_***a*** + ***Z***_2_***h*** + ***e***, (1)

***y*** is the vector of milk fat percentage records, ***b*** is the vector of fixed effects (lactation number, CDC, interaction of year–month by CDC), ***X*** is the design matrix that links the fixed effects to the milk fat percentage records, ***a*** is the vector of random animal effect, ***h*** is the vectors of random herd effects, ***Z***_1_ is the design matrices that link random animal effect to the fat percentage records, ***Z***_2_ is the design matrices that link the random herd effects to the fat percentage records, and ***e*** is the residuals.

The adjusted milk fat percentage records were used to obtain estimates of genetic parameters and GEBV using a random regression (RR) model executed using the Bayes R method.

y* = ***Z***_1_***a*** + ***Z***_2_***p*** + ***Z***_3_***h*** + ***e***, (2)

y* is adjusted milk fat percentage records, ***a*** = m + 1: additive genetic regression coefficients for each animal, ***p*** = m + 1: permanent environmental regression coefficients for each animal, ***h*** = m + 1: random regression coefficients for each herd, *m*: order of Legendre polynomial, ***e***: residuals, and ***Z***_1_, ***Z***_2_, and ***Z***_3_: incidence matrices of additive genetic, permanent environmental, and herd random regression coefficients, respectively.

The residual variance was assumed to be homogenous for milk fat percentage records within but heterogeneous between the lactation period classes. The assumed variance and covariance structures were as follows:

(3) $$var\left(\begin{array}{c} a\\ p\\ h\\ e \end{array}\right)=\left(\begin{array}{cccc} G⊗Ka & 0 & 0 & 0\\ 0 & I⊗Kp & 0 & 0\\ 0 & 0 & I⊗Kh & 0\\ 0 & 0 & 0 & R \end{array}\right)$$

where ***G***: genomic relationship matrix constructed using [34], ***Ka***, ***Kp***, and ***Kh***: the (co)variance matrices of the additive genetic, permanent environment, and herd regression coefficients, respectively, ⊗: direct product operation, ***I***: an identity matrix with dimensions equal to the number of levels of effects, and ***R***: diagonal matrix of residual variances. The model is estimated using MCMC (Markov chain Monte Carlo) with Gibbs Sampling in BayesRv2 with three parallel chains with 25,000 iterations, of which the first 5000 are discarded as burn-in and thinned by 10 (4000 MCMC samples per chain).

## 3. Results

The data collected on milk fat percentage traits on crossbred cows from various states were analyzed.

### 3.1. Phenotypic Quality Control

#### 3.1.1. Data Structure

**Figure 2:**
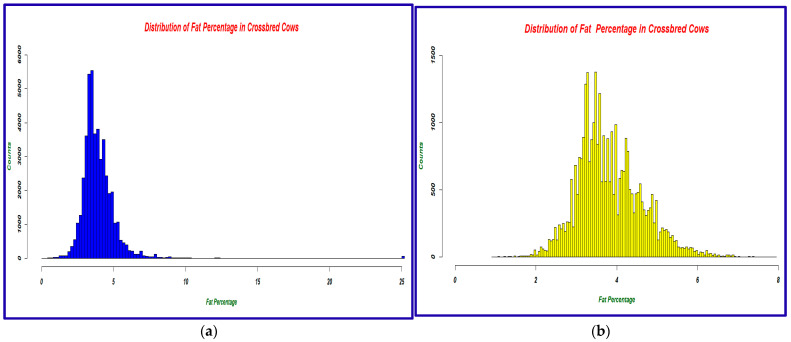
(**a**) Histogram of milk fat percentage data before quality control (**b**) Histogram of milk fat percentage data after quality control.

The quality control process resulted in retaining 33,845 milk fat percentage records from 1896 animals. The milk fat percentage in the raw data was summarized and visualized using a histogram before and after quality control (Figure 2).

#### 3.1.2. Herd Size and Number of Cows

**Figure 3:**
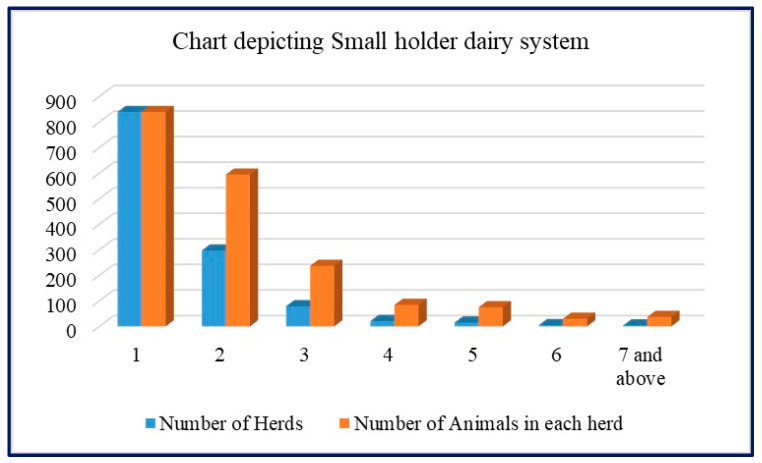
Chart depicting distribution of cows in smallholder dairy system.

The study examined the distribution of crossbred cows in herds, revealing a range of 1 to 14 cows per herd. The percentage distribution of crossbred cows within each herd size provided insights into the composition of farms in India (Figure 3).

The findings demonstrated that 44.23% of farms had a single crossbred cow, while 31.31% had two cows. Farms with three cows accounted for 12.49% of the total, while 4.43, 3.95, 1.58, and 2.0% represented the percentages for herd sizes of four, five, six, and more than seven cows, respectively. This evidence compellingly illustrates the wide prevalence of smallholder dairy systems in India (Table 1).

### 3.2. Genotypic Quality Control

The initial step taken for genotypic quality control involved the identification and removal of duplicate animals. However, upon thorough analysis, it was determined that there were no animals in the dataset that needed to be removed due to duplication. Subsequently, the dataset was scrutinized for animals that did not meet the required genotype call rate of 90 per cent. Only one animal fell below this threshold and was consequently eliminated from further analysis. Furthermore, the SNPs were examined to identify markers with low marker call rates and low MAF. A total of 4229 SNPs were found to have insufficient marker call rate or MAF and were consequently removed from the dataset. Following this filtration process, the remaining set of markers underwent screening to retain only those located in autosomes, resulting in the removal of 9089 markers that were not present in autosomes (Table 2). The resulting dataset of 1477 animals and 36,593 SNPs was deemed suitable for further analysis and research purposes.

### 3.3. Imputation

Before imputation, the squared correlation between the allele frequency among the HD 770K and GGP50K chips was measured at 0.594. However, after imputing the missing genotypes from the high-density chip, the squared correlation significantly increased to 0.882. This improvement indicates that the imputation process effectively enhanced the accuracy of the allele frequency estimation.

### 3.4. Residual Effects and Genetic Parameter

The residual effect of the individual fixed effects as well as the random effects of animals and herds were calculated, and the final adjusted records of a few individuals are presented in Table 3.

The adjusted milk fat percentage records ranged from −0.84 to 0.87. All the data were further utilized to find out the heritability. The variance and the heritability estimates of milk fat percentage traits in crossbreeds of HF and Jersey cattle under the smallholder dairy system are presented in Table 4.

### 3.5. Genomic-Estimated Breeding Value

The GEBV for milk fat percentage is given in Table 5. The highest GEBV recorded was 3.10%, while the lowest was −0.096%; the average GEBV was 0.054%.

The accuracy of calculating breeding values for milk fat percentage in crossbred dairy cattle was significantly improved by the genomic prediction models created in this study. Compared to conventional pedigree-based selection techniques, high-density SNP genotyping in conjunction with sophisticated statistical techniques like GBLUP and machine learning models produced higher predictive power. The results demonstrate that genomic selection can be a useful strategy for raising crossbreeds’ milk fat content, hence addressing a crucial nutritional and economic characteristic in the Indian dairy industry.

## 4. Discussion

The quality control process for our study involved collecting at least four milk fat percentage records per animal per lactation and removing outliers using the interquartile range. This approach was consistent with other studies conducted on different cattle populations [31,35,36]. In contrast, various studies were implemented with a minimum of five records for each milk production parameter per animal per lactation [37,38]. It becomes evident that, while our study and some others relied on a minimum of four records per animal per lactation, variations existed in the specific requirements for milk production records in different cattle populations and studies.

In line with our study, research on milk yield in crossbred cows with unknown levels of inheritance was conducted [31]. However, others ensured a fixed level of inheritance [39,40]. On the other hand, purebred populations were considered for this study based on their respective countries [35,36,37,38,41,42]. The data on milk fat percent recording was restricted from 8 to 340 days [31], 8–365 days [36,37,38,39,42], 5–305 days [42], and 4–500 days [32]. These differences can be attributed to the fact that previous studies were conducted in organized dairy farms, where daily records were maintained, in contrast to the test-day records used in our research. This broader range was relevant to their study focusing on smallholder dairy systems, where farmers often retain the animals until a significant decline in production or when they are ready to purchase new animals. The adoption of different data collection methods highlights the need for standardization in phenotypic quality control to enhance the reliability and comparability of research findings across diverse cattle populations and production systems.

The milk fat percentage data was included for further analysis only when it fell within three standard deviations. The final dataset in our study consisted of milk fat percentages ranging from 0.91 to 7.91. On par with our study, the fat percentages ranged from 1.5 to 8.5 [41] and 1.58 to 6.92 [43]. According to the number and noises in the dataset, four standard deviations were used as the criterion for milk production traits [31,44]. The age at the first calving was within the range of 18–36 months, which was set for the quality control process, aiming to maintain data integrity and minimize any potential inconsistencies or biases related to cow age and calving intervals [32,36,38,41,42]. However certain studies used de-regressed phenotypic records for the analysis of milk production traits to mitigate potential biases or confounding factors that could affect the accuracy of estimated breeding values [40,45,46,47].

In genotypic quality control, the individual call rates threshold was kept at 0.9 to exclude individuals with low genotyping completeness. This call rate was chosen since the genotyping was performed using the Illumina platform, where the error rate was minimal [38,39,45]. A high individual call rate was essential to maintain genetic analyses’ accuracy and statistical power. Individuals with low call rates may introduce bias or might compromise the quality of downstream analyses, such as genomic evaluations. Low call rates can be indicative of technical issues during genotyping, sample degradation, or inadequate DNA quality. Similar to that of individual call rates, SNP call rates were also kept at 0.95 [47], while 0.90 was used by several other researchers [3,7,32,38,42,45]. A high SNP call rate is crucial for accurate genetic analyses and the interpretation of the results. Low call rates may indicate genotyping failures, technical errors, or poor SNP performance. Such SNPs with low call rates can introduce noise, affect downstream analyses, and compromise the reliability of genetic association studies, genomic evaluations, or other analyses. Filtering SNPs based on MAF helps to focus the analysis on variants that are more informative and biologically relevant. The threshold for the MAF was kept at 0.01 in our study so as to retain SNPs with sufficient allele diversity for downstream analysis [31,38,47]. However, a lower threshold was kept at 0.02 [32,45] and 0.05 [42,46]. The moderate level of MAF ensured the retention of sufficient allele diversity for further analysis. By filtering out low-MAF SNPs, researchers can focus on more common genetic variants that are likely to have larger effects on the traits under investigation. A higher threshold may exclude potentially informative rare variants, while a lower threshold may increase the risk of including noise or artefacts in the analysis. By selecting a moderate MAF threshold, our study aimed to retain sufficient allele diversity while minimizing the inclusion of SNPs with low informativeness. This balance is crucial for achieving robust genetic analyses, particularly in population genetics, genomic prediction, and GWAS studies.

The removal of sex chromosome markers was a crucial quality control step in genetic studies [38,42]. This step was necessary because sex chromosomes, such as the X and Y chromosomes, differ in their inheritance patterns and genetic characteristics compared to autosomes. Including sex chromosome markers in the analysis could introduce bias and complicate the interpretation of results. For example, the presence of dosage compensation mechanisms in the X chromosome could lead to different gene expression patterns in males and females. Moreover, the Y chromosome was unique to males and contained genes that were not present in females.

The imputation successfully filled 44.84% of the missing data from the Illumina 50k chip with the Illumina 770k HD genotype, resulting in a dataset of 712,855 SNPs on autosomes for further analysis. The effectiveness of FImpute software Version 2.2 for imputing genotypes from low-density chips to high-density chips were reported with high accuracy in imputation, which allowed for more precise genomic analysis and improved data quality, enabling reliable predictions in genetic analysis [32,40,47,48,49,50,51,52].

The heritability of milk fat percentage was found to be 0.10 ± 0.036 among the smallholder dairy system in India. Low to moderate heritability estimates, ranging from 0.07 to 0.25 for different lactations in Thai crossbred animals, were reported [53]. On the other hand, a moderate heritability estimate of 0.31 was reported in Thai dairy cattle and Russian black and white cattle [42]. In contrast, a higher heritability for fat percentage was also recorded in certain dairy cattle across the globe [54,55,56,57]. These studies demonstrate the variability in heritability estimates for milk fat percentage in different cattle populations. The heritability estimates indicated the proportion of phenotypic variation attributed to genetic factors. The heritability of milk fat percentage was low, meaning that the variation in milk fat percentage among dairy cattle was due to environmental factors. The variation in heritability estimates across different studies indicates that customized breeding programs, tailored to specific crossbred populations, are necessary to improve milk composition effectively. Understanding the heritability of milk fat percentage in crossbred cattle is crucial for designing effective breeding strategies in India. While moderate to high heritability estimates are promising, addressing challenges related to data accuracy, infrastructure, and environmental influences is essential to realize genetic improvements in milk composition.

In the present study, the GEBV ranged from −0.096 to 3.10% with an average of 0.054%. In contrast to the present study, the GEBV of milk fat percentage in Holstein Friesian cattle ranged between −0.012 and 0.015%, with an average of 0.001% [45]. In another study (investigating the GEBV using single step random regression test-day model) for milk fat percentage in Thai dairy cattle found that the genetic potential ranged between −0.008 and 0.011%, with an average value of 0.003% [42]. On the other hand, the range of GEBV varied between −0.55 and 0.69% for cows and between −0.97 and 0.73% when sires were considered [25]. This suggests that, on average, the genetic potential for milk fat content in the studied population was relatively close to the baseline, indicating a limited deviation from the average performance. The animals with the best GEBV could be used for selection to accelerate the improvement of milk fat percentage.

The majority of prediction models in genomic selection are mainly concerned with additive genetic effects. But the amount of milk fat is also influenced by non-additive genetic influences like dominance and epistasis, especially in crossbred populations, where heterosis (hybrid vigor) is a concern. According to Misztal et al. [58], the existence of non-additive genetic variation implies that adding dominance and epistatic interactions to genomic prediction models could increase the precision of breeding value assessment. Studies have demonstrated that taking into consideration non-additive factors might enhance genetic assessments and selection tactics, particularly in varied crossbred populations, despite classic models’ assumption that additive effects are the main drivers of genetic merit [59]. Prediction accuracy can be further increased by combining genetic data with pedigree information. Better genetic evaluations for animals with incomplete genomic information can be ensured by including all available animals (both genotyped and non-genotyped individuals) into the study. This allows for a more accurate estimation of the connection matrix. In smallholder dairy systems, where full-genotyping herds might not be financially viable, this method is especially helpful. By utilizing data from their genotyped relatives, pedigree-based associations can assist in estimating breeding values for non-genotyped animals, thereby expanding the advantages of genomic selection to a larger population [60,61].

The prediction of GEBV for sires is an important step in using genomic selection to increase milk fat content. Because sires contribute genetics to numerous offspring, reliable GEBV estimates guarantee that only genetically superior bulls are employed in artificial insemination (AI) programs. In this study, GEBV forecasts for sires revealed significant diversity in genetic merit for milk fat percentage, highlighting the significance of genomic selection in selecting high-value breeding stock. The use of genomic information eliminated the need for progeny testing, which is time-consuming and expensive, allowing for faster genetic development in smallholder dairy systems [25,60]. Furthermore, the use of genomic selection in sire evaluation allows for a more precise selection of bulls suitable for various breeding purposes. Farmers who prioritize high-fat milk production can choose sires with the highest GEBVs for milk fat percentage, whereas those who prioritize balanced milk yield and composition can select bulls with optimum genetic profiles. Integrating genomic selection into national dairy breeding programs has the potential to expedite genetic improvement in crossbred cattle, resulting in enhanced profitability and sustainability in smallholder dairy farming [25,62].

The current study is of vital importance to all parties involved in cattle rearing. However, the great variety of environmental stressors (SSI, heat stress, husbandry, and food conditions) complicates statistical modeling methods. Furthermore, in tropical nations, genotyping and phenotyping remain difficult, signaling sample size constraints. As a result, the relatively large SE associated with genetic and genomic parameter estimations is prevalent in studies conducted in developing nations. Nonetheless, the findings of this study encourage further research in tropical nations, taking into account genetic pathways in the context of environmental constraints.

The present findings have important practical implications for smallholder dairy farmers. Because smallholders frequently face resource limits, enhancing milk composition through selective breeding can improve both market value and household nutrition. Higher milk fat content boosts the profitability of milk sold in local marketplaces, where customers favor rich, creamy milk for direct consumption and traditional dairy products like ghee and paneer. Furthermore, boosting milk fat content by genetic selection enables farmers to earn higher returns without necessarily increasing milk supply, which is especially essential for those with limited access to quality feed and veterinary care.

The main limitation of this study lies in the accuracy of phenotypic and genotypic data collection, which is crucial for effective genomic selection. However, in India’s smallholder dairy systems, gathering such data presents significant logistical challenges. Additionally, establishing the infrastructure required for large-scale genotyping and data analysis demands substantial investment and technical expertise, both of which are still in their early stages. Given India’s diverse cattle breeds, developing breed-specific genomic selection strategies is essential to ensure precision and effectiveness. Encouragingly, the National Dairy Development Board has recently initiated efforts in this direction, signaling promising advancements in selecting breeds with specific desirable traits. The National Dairy Development Board’s recent initiatives in genomic breeding provide an optimistic outlook, suggesting that, with continued investment and improved data infrastructure, genomic selection could become a key driver of dairy productivity enhancement in India.

## 5. Conclusions

This study found that an average herd size of one or two animals accounts for 75.54 percent, which is consistent with the smallholder dairy system in India. A total of 12,133 milk fat percentage data were eliminated during the phenotypic quality check and, hence, digitalized entry must be performed at the doorstep to remove the bias from the recording. This study discovered that genotyping may be accomplished using a 50K SNP chip, which is cost-effective. This approach enabled more comprehensive and precise genetic predictions. The inclusion of indigenous breeds in the reference population will help accurate genomic prediction. Even though the heritability and GEBV recorded in the study population were low, the technique could be used with caution to select the animal for improving milk fat percentage among the crossbred cows in the smallholder dairy system in India. These findings add to the body of knowledge for researchers and policymakers, particularly in initiating further studies in India or other tropical regions to explore the mechanisms of selection and genomics in the context of environmental changes. Given that the study is constrained by a relatively small sample size, it is crucial to validate the results through ongoing genomic analyses, especially in tropical breeds raised under challenging environmental conditions. Given India’s diverse dairy cattle populations and the dominance of smallholder dairy farming, the integration of genomic selection tools with real-time performance monitoring and strategic crossbreeding programs offers a sustainable path for improving milk fat percentage.

## Figures and Tables

**Table 1:** Number of herds and crossbred cows in each herd size.

Herd Size	Herds	Number of Animals
1	839	839
2	297	594
3	79	237
4	21	84
5	15	75
6	5	30
7 and above	4	37
Total	1260	1896

**Table 2:** Summary of genotypic quality control process.

Conditions	Threshold	Number of Animals/SNPs Screened	Number of Animals/SNPs Retained	Number of Animals/SNPs Dropped
No. of duplicates removed	0.98	1478	Nil	Nil
No. of animals’ call rates	0.90	1478	1477	1
No. of markers with low call rates and low MAF removed	0.95 0.01	49,911	45,682	4229
No. of markers only in autosomes	Removal of X, Y, MT chromosome markers	49,911	40,822	9089

**Table 3:** Adjusted milk fat percentage records of a few individuals.

Sl.No.	Animal ID	IFE	ARE	HRE	AFP
1	6BA9CA3D-B98E-40DC-B86A-0B2116127746	0.13	−0.05	0.05	0.13
2	6BA9CA3D-B98E-40DC-B86A-0B2116127746	−0.10	−0.05	0.05	−0.11
3	6BA9CA3D-B98E-40DC-B86A-0B2116127746	0.44	−0.05	0.05	0.44
4	6BA9CA3D-B98E-40DC-B86A-0B2116127746	0.49	−0.05	0.05	0.49
5	013CFDC6-0446-471B-AE0D-545834C921AB	−0.33	0.30	0.05	0.03
6	013CFDC6-0446-471B-AE0D-545834C921AB	0.44	0.30	0.05	0.79
7	6BA9CA3D-B98E-40DC-B86A-0B2116127746	0.87	−0.05	0.05	0.87
8	6BA9CA3D-B98E-40DC-B86A-0B2116127746	−0.84	−0.05	0.05	−0.84
9	013CFDC6-0446-471B-AE0D-545834C921AB	−0.30	0.30	0.05	0.05
10	6BA9CA3D-B98E-40DC-B86A-0B2116127746	0.28	−0.05	0.05	0.28

IFE—Individual fixed effect, ARE—Animal residual effect, HRE–Herd residual effect, AFP—Adjusted fat percentage.

**Table 4:** Variance component and heritability estimates of milk fat percentage.

Parameter	Milk Fat Percentage
Additive genetic variance (V_a_)	0.012
Environmental variance (V_e_)	0.106
Total phenotypic variance (V_p_)	0.118
Heritability (h^2^)	0.10 ± 0.036

**Table 5:** GEBV of a few animals for milk fat percentage.

Animal ID	GEBV (%)
0BB54BC5-F4E4-45C0-903A-A7552588CB13	3.10
1FAA4E0A-75DA-44D5-A94A-A9D7E0252CD6	2.77
3A324D0B-E441-4D59-948D-302CF3FCCCBF	2.44
6ACFB5B6-A14D-403B-9331-8CD0E740439B	2.32
8A3022D7-53F1-4691-A555-EBA1FB5DBC16	2.21
5D127AF5-80CE-4353-B146-401623836E0F	1.63
60AA4C28-B1C4-4AFE-B353-A33327FDE7B0	1.63
87CB327A-C7D6-4DE4-9B99-4E07E94233D5	1.14
2079EDB5-4B26-496E-8CAD-C24F19DB292E	1.11
5A7911D7-F0A6-48D7-9028-1145FE2BCEAE	1.07
6E9BED8F-964B-44FC-AC2C-0CE95A8A12B8	1.05
2996D785-6FD9-42EC-96C0-CF9B59A5E13E	1.05
293DC3D5-9DBE-4C6E-A48E-F645FD5646D8	1.00
7ADB65B4-69AB-4D0C-B460-A0595833BD94	0.97
73BEA21E-6DB6-47FA-A69B-B344797F7126	0.95
405BA838-B753-417D-8B0F-C78D484EF8DF	0.22
82B60872-60DC-450E-A10D-125A87423BB2	0.21
46569474-EF61-40BF-A59C-C66CE550E423	0.13
0D471917-2B6B-4207-90EE-B2655C4FF5DE	0.12
405BA838-B753-417D-8B0F-C78D484EF8DF	0.22
08D7934D-2CBB-44E2-B9C4-85F0E7C9CA84	−0.095
5F8C5E44-918B-4FBF-816F-80B6FC9F1B38	−0.096
2FDCDC60-0FC3-4815-B32C-7DF1A95CF677	−0.096
4AF62135-6568-4262-A994-C82815927CD7	−0.096
1F590D1D-C358-4F6F-B97D-7D580C9D93FD	−0.096

GEBV—Genomic-estimated breeding value.

## Data Availability

The dataset created and analyzed in the current study will be made available upon reasonable request.
